# Crossover from polariton lasing to exciton lasing in a strongly coupled ZnO microcavity

**DOI:** 10.1038/srep20581

**Published:** 2016-02-03

**Authors:** Ying-Yu Lai, Yu-Hsun Chou, Yu-Pin Lan, Tien-Chang Lu, Shing-Chung Wang, Yoshihisa Yamamoto

**Affiliations:** 1 Department of Photonics, National Chiao Tung University, Hsinchu 300, Taiwan; 2 E. L. Ginzton Laboratroy, Stanford Univeristy, Stanford CA, 94305, USA; 3 National Institute of Informatics, Hitotsubashi, Chiyoda-ku, Tokyo 101-8430, Japan

## Abstract

Unlike conventional photon lasing, in which the threshold is limited by the population inversion of the electron-hole plasma, the exciton lasing generated by exciton-exciton scattering and the polariton lasing generated by dynamical condensates have received considerable attention in recent years because of the sub-Mott density and low-threshold operation. This paper presents a novel approach to generate both exciton and polariton lasing in a strongly coupled microcavity (MC) and determine the critical driving requirements for simultaneously triggering these two lasing operation in temperature <140 K and large negative polariton-exciton offset (<−133 meV) conditions. In addition, the corresponding lasing behaviors, such as threshold energy, linewidth, phase diagram, and angular dispersion are verified. The results afford a basis from which to understand the complicated lasing mechanisms in strongly coupled MCs and verify a new method with which to trigger dual laser emission based on exciton and polariton.

A microcavity (MC), which confines photons in a wavelength scale resonator, has numerous applications, such as in vertical-cavity surface-emitting lasers, single quantum emitters, and other optoelectronic devices, depending on the active layer design^1^,^2^,^3^,^4^,^5^,^6^. Because the active layer in the cavity exhibits excitonic behavior, the MC can enter a strong exciton-photon coupling regime and generate a hybrid quasiparticle called polariton^7^,^8^. Within a strongly coupled MC, the polariton laser—a novel type ultralow threshold coherent light source emitted from a condensed polariton state that does not require the electron-hole plasma to be in a population inversion condition—has been reported in various types of MC^9^,^10^,^11^,^12^,^13^,^14^,^15^,^16^,^17^,^18^. In addition to polaritons, the so-called *P-band,* generated through exciton-exciton inelastic scattering (X-X) mechanisms in the phase-space at an intermediate exciton density, can generate a high excitonic gain to compensate the loss to achieve the lasing action^19^,^20^,^21^,^22^,^23^. These two exciton-related lasing actions greatly influence future ultralow threshold lasers and should exhibit some correlation. They can also be manipulated since they share the same exciton reservoir. However, these two lasing actions have not been simultaneously triggered or studied in strongly coupled MCs because of critical operation condition, which involves difficulty encountered when attempting to obtain adequate exciton numbers to generate excitonic gain in a strongly coupled MC. Considering this, ZnO, which exhibits strong excitonic behavior, including large exciton binding energy and oscillator strength, can possibly achieve excitonic P-band lasing in a strongly coupled MC. Although the polaritonic behavior and low-threshold polariton lasing in a ZnO MC have been reported, simultaneous excitonic P-band lasing and polariton lasing in a ZnO MC remain unreported^24^,^25^,^26^,^27^,^28^,^29^,^30^,^31^,^32^,^33^,^34^,^35^,^36^. In this study, we investigated polariton lasing and P-band lasing in a strongly coupled ZnO MC. The polariton lasing can be maintained up to 353 K because of the robust exciton properties in ZnO. The conditions required to simultaneously trigger these two lasing actions were specified by the operating temperature and lower polariton branch (LPB)-exciton offset (Δ = *E*_*LPB*_ *− E*_*x*_, where *E*_*LPB*_ and *E*_*x*_ represent the energy of LPB and exciton, respectively). The experimental investigations indicate that the P-band exciton lasing was effective when the negative LPB offset was large (|Δ|>133 meV). However, achieving polariton lasing in this offset is difficult because of the relatively short lifetime of the polariton. The P-band exciton lasing can coexist with polariton lasing in a moderate pumping condition.

## Results

### Mechanism illustration

**Figure 1:**
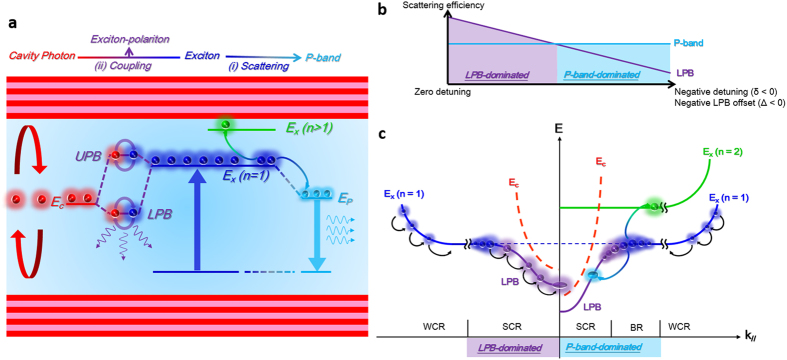
Schematic illustrations of exciton transition channels in a strong coupled ZnO MC. (**a**) Excitonic-related lasing mechanisms in the strongly coupled ZnO MC. Different states in a strongly coupled ZnO MC including photons (red), ground state excitons (navy blue), higher state excitons (green), exciton-polaritons (blueviolet), and P-band exciton (sky blue) are represented by different colors. The P-band exciton is formed through the X-X scattering such that one of the exciton would scatter to the higher energy state (n > 1) and another one scatters to a lower energy level (P-band) under energy and momentum conservation. (**b**) Schematic of scattering efficiency trade-off between P-band and polaritons versus the negative detuning and LPB-offset of cavity. The exciton-exciton scattering is independent of the detuning parameter but increases with the exciton density, while the polariton scattering decreases with the detuning parameter. (**c**) Schematic of the dispersions of a small negatively detuned MC (Left side) and a large negatively detuned MC (Right side). The SCR, WCR and BR indicate the strong coupling region, weak coupling region, and bottleneck region respectively.

Figure 1a shows a simple schematic plot of two exciton-related lasing mechanisms in a strongly coupled and negatively detuned ZnO MC. Excitons (navy blue spheres) are first generated from the excitation. There are two exciton transition channels: (i) X-X scattering, or the P-band (sky blue spheres); and (ii) exciton-polariton emission (blue-violet circles) formed by coherent coupling between the excitons and cavity photons (red spheres). During X-X scattering, one exciton scatters to a high energy state (n > 1) and the other scatters to a low energy level (P-band) under energy and momentum conservation. Because the injected exciton population is limited, the two channels compete with each other, resulting in a complicated nonlinear transition behavior. The polaritons within the MC could scatter with different excitations, such as polaritons, phonons and electrons, because of their exciton (matter) part. Therefore, the polariton lifetime plays a crucial role in its scattering probability (efficiency). In the ZnO MC, the bare cavity photon lifetime calculated using the measured *Q* value was 0.39 ps. The cavity detuning (*δ* = *E*_*c*_ *− E*_*x*_, where *E*_*c*_ is the cavity photon energy and *E*_*x*_ is the exciton energy) was tuned from 0 (50% exciton fraction) to −150 meV (11% exciton fraction) by selecting a proper pumping position on the MCs. Thus, the polariton lifetime could range from 0.78 ps (50% exciton fraction) to 0.43 ps (11% exciton fraction) as the detuning decreased from 0–150 meV (Supplementary Information, Part I). By contrast, the P-band lifetime was independent of the cavity detuning because it was generated from the exciton reservoir without cavity photons. Thus the lasing mechanism from the two channels in the strongly coupled ZnO MC could be easily manipulated by adjusting the cavity detuning. Figure 1b illustrates a simple sketch of the variation in scattering efficiency versus the cavity detuning and LPB-offset in the strongly coupled ZnO MC. The sky-blue line and blue-violet line represent the P-band exciton and polariton, respectively. When detuning was zero, the polariton lifetime was long enough to exhibit a more efficient stimulated scattering event than that of the P-band because of a higher exciton fraction. The polariton lifetime and scattering efficiency shortened and lowered as the cavity became more negatively detuned. The intersection point shown in Fig. 1b depicts the transition of scattering efficiency between polaritons and P-band. For the case that the value of negative detuning (or LPB-offset) is smaller than that intersection point, the emission is dominated by the polariton and the corresponding polariton lasing can be achieved through the polariton scattering, as shown in the left side of the Fig. 1c. Once the value of negative detuning is larger than that crossing point, the poor polariton scattering would results in a bottleneck effect in which polaritons would accumulate at high energy/momentum state, as shown in the BR of the Fig. 1c. The more efficient P-band scattering will dominates the stimulated scattering process and corresponding lasing action. Thus, the lasing action in a strongly coupled MC could switch from polariton to P-band exciton and be manipulated by selecting appropriate detuning conditions.

### Characteristics of polariton lasing threshold at temperature above 140 K

**Figure 2:**
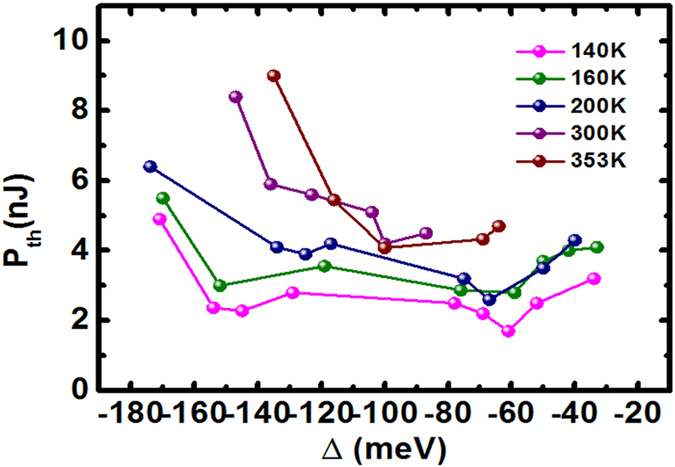
Measured polariton laser threshold vs. Δ in a strongly coupled ZnO MC. Polariton lasing threshold versus LPB-exciton offset at the temperature ranging from 140 K to 353 K. The shallow orange section and green section represent the region of the first and second LO phonon replica.

The phase diagram is commonly used to investigate the kinetics and thermal dynamics of polaritons^37^,^38^,^39^,^40^. We verified the basic lasing mechanisms by investigating the simplified phase diagram depicted in Fig. 2, which shows the threshold evolution versus the LPB-offset for temperature ranging from 140 K to 353 K. In this temperature region, P-band was not observed in our measurement due to the stronger thermal broadening which would significantly weaken the X-X scattering efficiency. For temperatures lower than 200 K, the threshold tendency indicated two minima, located at approximately −70 meV and −140 meV offsets. These two optimal offsets corresponded to the efficient first and second order longitudinal-optical (LO) phonon-assisted polariton relaxation, which can assist the polariton stimulated scattering to the final state^41^,^42^. (Refer to Part II of the Supplementary Information for the corresponding simulation.) The second order LO phonon-assisted polariton relaxation extended the range of polariton lasing to a more negative cavity detuning. When the temperature exceeded 200 K, the phonon-assisted behavior was moderated by thermal broadening. This property is verified by our simulation described in Part II of the Supplementary Information. According to the results depicted in Fig. 2, the low-threshold polariton lasing can be achieved across a wide cavity detuning range and at high temperatures (even above 350 K, refer to Part IV of the Supplementary Information), primarily because of the large oscillator strength and exciton binding energy of ZnO, which maintains stable polaritonic operation, and the efficient relaxation of polariton through LO phonon scattering.

### Lasing characteristics of small and moderate negative-detuned MCs at temperature below 140 K

**Figure 3:**
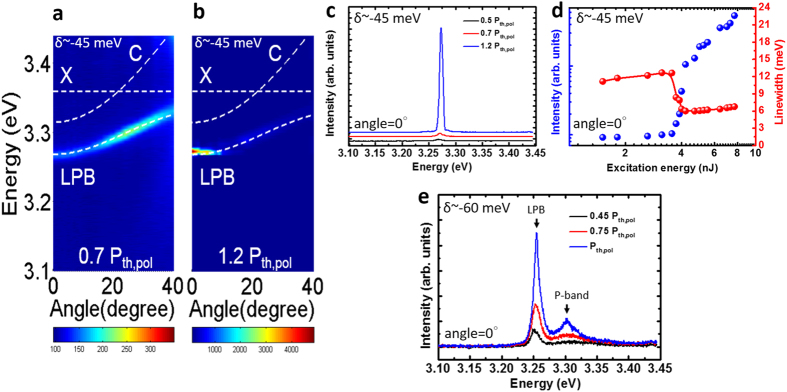
Lasing properties of the small negative detuned strong coupled ZnO MC. (**a**,**b**) Angle-resolved photoluminescence mapping at 0.7 and 1.2 times of the polariton lasing threshold with a negative detuning of −45 meV at 100 K. (**c**) Measured emission spectra recorded between 0.5 and 1.2 times of the polariton lasing threshold with a negative detuning of −45 meV at 100 K at the detection angle = 0°. (**d**) Peak intensities and linewidths of the polariton emissions versus excitation energy with a negative detuning of −45 meV at 100 K at the detection angle = 0°. (**e**) Measured spectra at a larger negative detuning case (δ = −60 meV) at 100 K at the detection angle = 0°. The dashed lines in (**a**,**b**) indicates the exciton mode (X), cavity mode (**c**), and LPB.

The exciton signature was more substantial when the temperature was lower because the thermal broadening was suppressed. Figure 3a,b show the angle-resolved photoluminescence (ARPL) mappings below and above the polariton laser threshold at 100 K, indicating a clear polariton emission accompanied with an anti-crossing feature when excitation was low. The corresponding cavity detuning was −45 meV (LPB-exciton offset Δ approximately −91 meV) and the Rabi splitting was 130 meV. When excitation increased, the polaritons began to lase with no other emissions as shown in Fig. 3c. The LPB emission in Fig. 3c shows a nonlinear increase and a slight blue-shift caused by the polariton-polariton interaction when the excitation reached the threshold. The polariton lasing threshold (P_th,pol_) was 4 nJ in Fig. 3d, and the linewidth broadening reflects the influence of ground-state polariton self-interaction. When the cavity detuning becomes more negative (δ = −60 meV), as shown in Fig. 3e, another emission peak occurred at higher energy side. This additional emission was related to the P-band exciton, which was caused by the efficient X-X scattering when pumping conditions were moderate. In Fig. 3e, the P-band line appears in the ZnO MC emission when the detuning became more negative; however, the line was too weak and the polariton lasing still dominated. (Refer to Part IV of the Supplementary Information for detailed peak intensities versus excitation energy.) The corresponding emission peak energy of the P-band (3.302 eV) was equal to the *P*_*2*_ energy expressed in the following equation:


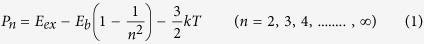


where *P*_*n*_ is the P-band energy, *E*_*ex*_ represents the free exciton emission energy, *E*_*b*_ is the exciton binding energy, *n* represents the quantum number of the envelope function, and *kT* is the thermal energy^13^. The occurrence of the P-band emission shows that |δ| and |Δ| are close to the intersection point shown in the Fig. 1b so that LPB and P-band can co-exist with each other.

### Lasing characteristics of large negative-detuned MCs at temperature below 140 K

**Figure 4:**
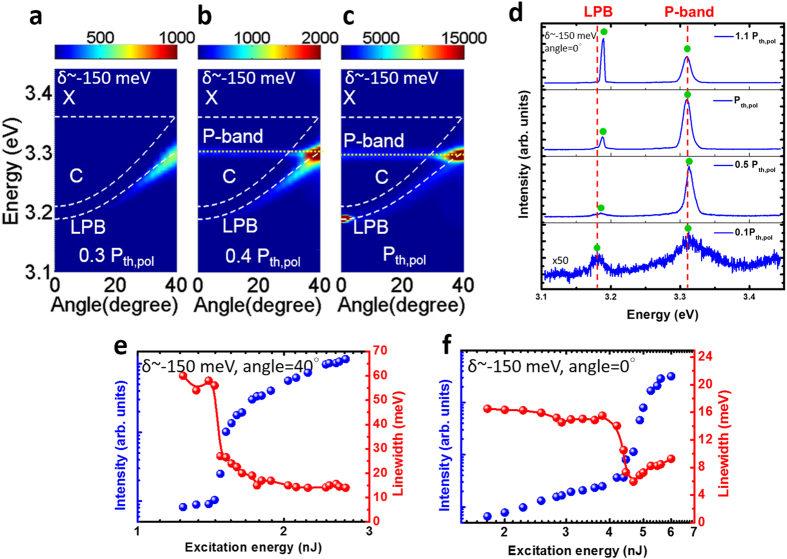
Lasing properties of the large negative detuned strongly coupled ZnO MC. (**a**–**c**) Angle-resolved photoluminescence mapping at 0.3–1 times of polariton lasing threshold with a δ of −150 meV at 100 K. (**d**) Measured emission spectra recorded between 0.1 and 1.1 times of polariton lasing threshold (Green spheres point out the peak location of P-band and polariton) with a δ of −150 meV at 100 K and the detection angle = 0°. (**e**) Peak intensities and linewidths of the P-band emissions versus excitation energy with a δ of −150 meV at 100 K and the detection angle = 40°. (**f**) Peak intensities and linewidths of the polariton emissions versus excitation energy with a δ of −150 meV at 100 K and the detection angle = 0°. The dashed lines in (**a**) to (**c**) indicate the exciton mode (X), cavity mode (c), and LPB. The dot lines in (**b**) and (**c**) depict the P-band exciton emission.

As the |δ| or |Δ| increased, the polariton relaxation efficiency decreased because of its shorter lifetime and low probability of scattering with other excitation. Thus, the emission was gradually dominated by the P-band exciton when the absolute value of LPB-exciton offset keeps increased because the polariton scattering decreased with the increasing offset. Figure 4a–c illustrate the cavity emission evolution operated at a large |Δ| from low to high excitation. The Δ was approximately −171 meV and the corresponding δ was nearly −150 meV. In this large negative detuned MC, the polariton emission was relatively weaker than the P-band exciton emission when the MC was operated at a low excitation of approximately 0.3 P_th,pol_ (threshold of polaritons) due to its short lifetime (0.43 ps) and inefficient scattering. As the pumping energy increased, the P-band began to lase at 0.4 P_th,pol_ and the intensity and linewidth simultaneously increased nonlinearly and narrowed. Moreover, the P-band energy was unrelated to the cavity detuning because it was purely determined by X-X scattering (Part III of the Supplementary Information). The polariton band began to lase and coexisted with the P-band lasing at threshold of P_th,pol_, as shown in Fig. 4c. Figure 4d depicted a more specific analysis of the emission spectra at a 0 degree collection angle. The LPB emission and P-band coexisted at 0.1 P_th,pol_. The P-band linewidth distinctly narrowed and the intensity nonlinearly increased at 0.5 P_th,pol,_ corresponding to the lasing action. Then the LPB starts to lase at P_th,pol_. Simultaneous lasing of the polariton and P-band was achieved at 1.1 P_th,pol_. Apparently, the LPB emission peak indicated an 8 meV blue shift from 0.1 P_th_ to P_th_, and remained far below from the cavity mode. This phenomena indicates that strong coupling persisted in the measurements even when the P-band was involved. On the contrary, the P-band emission exhibited nearly no energy shift when excitation increased. Figure 4e,f show the intensity and the linewidth of the P-band and polariton lasing versus excitation energy. The corresponding thresholds of P-band and LPB were 1.5 nJ and 4.8 nJ, respectively. Above the threshold, polariton lasing linewidth broadened because of its self-interaction. By contrast, the P-band lasing linewidth did not broaden.

**Figure 5:**
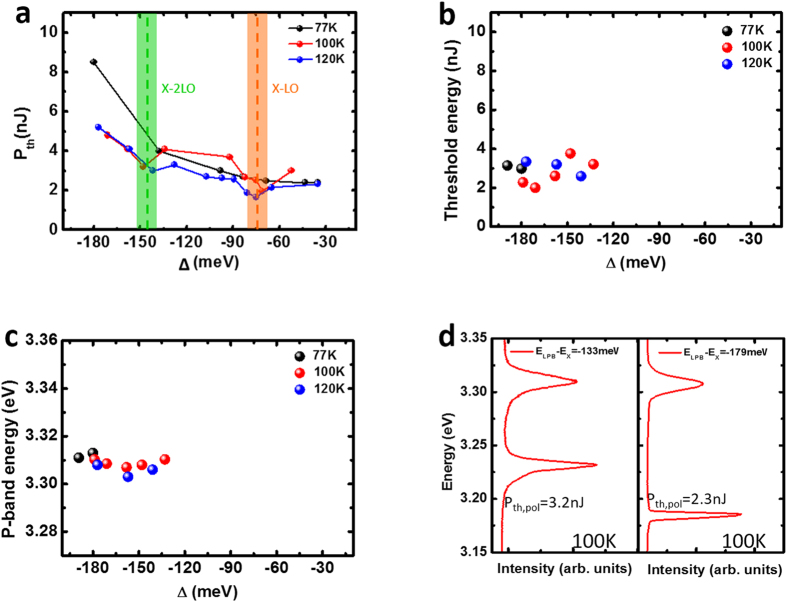
Properties of polariton and P-band lasing. (**a**) Measured polariton laser threshold vs. **Δ** at temperature ranging from 77 K to 120 K. (**b**) Measured P-band laser threshold vs. **Δ** and (**c**) P-band energy vs. **Δ**. (**d**) Spectra of simultaneous polariton/P-band lasing at **Δ** = −133/−179 meV LPB-exciton offset and their energy gap. The shallow orange section and green section in (**a**) represent the region of the first and second LO phonon replica.

In addition, we investigated the simplified phase diagram of the polariton and P-band when temperatures ranged from 77 K to 120 K, which are shown in Fig. 5a,b. The polariton lasing threshold shows a double minimum feature at proper offsets, which is caused by the efficient polariton-LO phonon scattering. The polariton lasing threshold also suggest an overall increasing tendency with increasing |Δ| due to the decreasing of excitonic portion. P-band lasing was only observed at large offset (exceeding than −133 meV) in which the polariton scattering was relatively inefficient (Fig. 5b). The P-band lasing thresholds were approximately 3 nJ and did not strongly depend on the temperature and detuning. Therefore, the P-band lasing threshold was lower than that of the polariton lasing at large negative offset. Furthermore, the polariton lasing at certain offsets exhibited higher thresholds at low temperature than at high temperature because of competition for the exciton reservoir by P-band lasing. At 100 K, the offset parameter that simultaneously triggered the polariton lasing and the P-band lasing ranged from −133 meV offset to −179 meV. Figure 5c shows the spectra of the polariton lasing and P-band lasing at simultaneously triggered condition.

In summary, we determined the condition in which to simultaneously trigger exciton-related polariton lasing and P-band lasing in the ZnO MC. In addition, we verified the characteristics of the lasing behavior. The strongly coupled polariton lasing had a double optimum among the exciton-LPB offset due to the LO-phonon assisted relaxation at temperatures ranging from 140 K to 353 K. For lower temperature (below 140 K), another laser emission, P-band, simultaneously occurred with a lower threshold than the polariton when the exciton-photon detuning was lower than −133 meV. The energy offset between the P-band lasing and polariton lasing could be tuned to 38 meV. This study affords a guide for controlling the two sub-Mott density, and exciton-related lasing mechanisms, and has a high potential for future low-threshold lasers and other polaritonic physics development.

## Methods

The sample was grown on a (0001) oriented sapphire substrate. The bottom 30-pair epitaxial AlN/AlGaN distributed Bragg reflector (DBR) was initially grown using metal-organic chemical vapor deposition (MOCVD). The GaN/AlN super lattice (SL) layers were inserted in every 3–5 AlN/AlGaN DBR pairs during the growth process to suppress the tensile strain in the DBRs and achieve high reflective epitaxial DBRs with wide stop-band width. The ZnO 3λ/2-thick cavity was then grown on the AlN/AlGaN DBR by using pulsed-laser deposition. The thickness gradient of the growth provided a series of cavity detunings, facilitating experimental measurements. Finally, the sample was completed after 9-pair SiO_2_/HfO_2_ dielectric DBR was deposited.

The photoluminescence (PL) measurements were pumped using a third harmonic generation (THG) of Nd:YVO_4_ pulse laser with a 0.5 ns pulse duration. The PL could be divided into two setups for different purpose: ARPL and micro-PL. The pumping laser was inclined-incident and focused to around 30 μm in radius onto the sample by using a lens with a 10-cm focus length in the ARPL system. The cavity emission was collected using a UV optical fiber mounted on a rotating stage that had an angular resolution of 1°, and detected by a nitrogen-cooled charge-coupled device. This setup was more effective for large negative detuning than was the commonly used Fourier imaging technique, because the collection angle involved in Fourier imaging is limited by the numerical aperture (NA) of objective lens. The pump beam and cavity emission were focused and collected using a microscope objective lens with 0.55 NA. The focal spot of micro-PL was nearly 3 μm, enabling effective measuring of the sample’s local cavity *Q*-value.

## Additional Information

**How to cite this article**: Lai, Y.-Y. *et al*. Crossover from polariton lasing to exciton lasing in a strongly coupled ZnO microcavity. *Sci. Rep.* **6**, 20581; doi: 10.1038/srep20581 (2016).

## Supplementary Material

Supplementary Information
