# Supplementary material for: Crossover from polariton lasing to exciton lasing in a strongly coupled ZnO microcavity
Source: Sci Rep. 2016 Feb 3;6:20581. doi: 10.1038/srep20581 (PMC4738341; doi:10.1038/srep20581)
Supplement: Supplementary Information [file srep20581-s1.doc]

**Supplementary Information**

Crossover from polariton lasing to exciton lasing in a strongly coupled ZnO microcavity

**Ying-Yu Lai^1^, Yu-Hsun Chou^1^, Yu-Pin Lan^1^, Tien-Chang Lu^1*^, Shing-Chung Wang^1^ and Yoshihisa Yamamoto^2, 3^**

^1^Department of Photonics, National Chiao Tung University, Hsinchu 300, Taiwan.

^2^E. L. Ginzton Laboratroy, Stanford Univeristy, Stanford CA, 94305, USA.

^3^National Institute of Informatics, Hitotsubashi, Chiyoda-ku, Tokyo 101-8430, Japan.

*email: [timtclu@mail.nctu.edu.tw](mailto:timtclu@mail.nctu.edu.twR)

**I. Local Q value and lifetime analysis of ZnO microcavity**

Here, we present the local quality factor (Q) of ZnO microcavity (MC) using micro-photoluminescence (PL) measurement. It’s important to use micro-PL to get the local Q value since the ZnO MC is commonly suffered from the in-plane photonic disorder.^1^ The focal spot of micro-PL is about 3μm. The typical measured spectrum linewidth is about 5 meV which corresponds to a Q value of about 660, as shown in Fig. S1. Some selected points can achieve a Q value of about 1500. Compared to those shown in Fig. 3d and Fig. 4f of the main manuscript, the linewidth measured by micro-PL setup exhibits a higher Q value due to the smaller pumping spot.


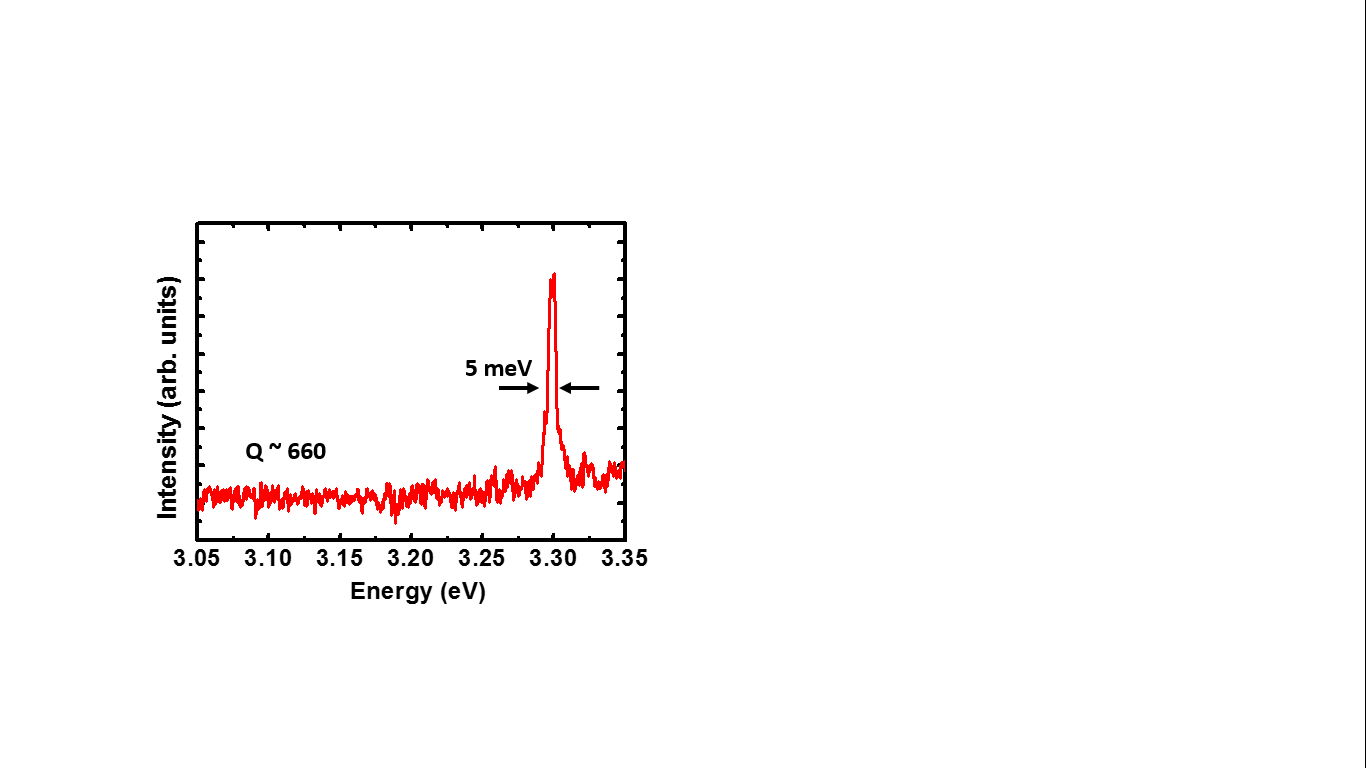


**Supplementary Figure S1.** Emission spectrum of the ZnO MC measured by the micro-PL setup.

From Fig. 1 in the main manuscript, the total polariton scattering efficiency including polariton-polariton, polariton-phonon scattering would be highly related to the cavity-exciton detuning, which determines the polariton lifetime as the following equation,

$\frac{1}{\tau_{p}}=\frac{\left|x\right|^{2}}{\tau_{x}}+\frac{\left|c\right|^{2}}{\tau_{c}}$ (1)

where τ_p_, τ_c_, and τ_x_ stand for the lifetime of polaritons, cavity photons, and excitons. $\left|x\right|$^2^ and $\left|c\right|$^2^ depict the fraction of exciton and cavity photon of a composed polariton. The cavity lifetime calculated from the local cavity Q value mentioned above is about 0.39 ps and the exciton lifetime is set to be 300 ps.^2, 3^ From the measurement, cavity detuning could be adjusted from 0 to -150 meV with corresponding exciton fraction ranging from 50 % to 11 %. The resulted polariton lifetime is 0.78 ps for the zero detuning case and 0.43 ps for the -150 detuning case.

**II. Simulation of threshold dependence of polariton condensate**

The threshold dependence of polariton condensate was modeled by the semi-classical Boltzmann equation.^4^ At first, two excitons in the reservoir will scatter with an initial energy $\varepsilon_{i}^{x}$ and wavevector $k_{i}^{x}$. One of the exciton will scatter to a higher state with a final energy $\varepsilon_{f}^{x}$ and wavevector $k_{f}^{x}$ and another into the LPB with energy$\varepsilon_{k}^{lp}$. By neglecting the small wavevector of the LPB, the energy/wavevector conservation relation can be expressed as :

$\begin{matrix} 2\left|k_{i}^{x}\right|=\left|k_{f}^{x}\right|\to4\varepsilon_{i}^{x}=\varepsilon_{f}^{x}, \\ 2\varepsilon_{i}^{x}=-\left|\varepsilon_{k}^{lp}\right|+\varepsilon_{f}^{x}\to\varepsilon_{i}^{x}=\left|\varepsilon_{k}^{lp}\right|/2,\varepsilon_{f}^{x}=2\left|\varepsilon_{k}^{lp}\right|. \end{matrix}$ (2)

which means that two excitons need an energy $\left|\varepsilon_{k}^{lp}\right|/2$ to be the initial states of a process in which one of them falls into the LPB. The corresponding rate equation of polaritons and excitons are given by equations (3) and (4).

$\frac{dN_{k}^{lp}}{dt}=\left(1+N_{k}^{lp}\right)\left(W_{k}^{in}n_{x}^{2}+\sum_{k'} W_{k\to k'}^{lp-LO}N_{k'}^{lp}\right)-N_{k}^{lp}[W_{k}^{out}n_{x}+\sum_{k'} W_{k\to k'}^{lp-LO}\left(1+N_{k'}^{lp}\right)]-\Gamma_{k}^{lp}N_{k}^{lp}$ (3)

$\frac{dn_{x}}{dt}=-\frac{1}{S}\sum_{k} dg_{k}^{lp}\left[W_{k}^{in}n_{x}^{2}\left(1+N_{k}^{lp}\right)-W_{k}^{out}n_{x}N_{k}^{lp}\right]-\Gamma n_{x}+p_{x}$ (4)

where *S*, *P_x_*,
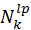
, *dg_k_^lp^*, and
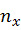
 are the quantized area, exciton pumping rate, occupation numbers in the lower polariton branch, degeneracy of polariton level in *k* state and exciton reservoir density, respectively. The polariton decay rate *Г_k_^lp^* is defined by:

$\Gamma_{k}^{lp}=\frac{1}{\tau_{p}}$ (5)

where the polariton lifetime *τ_p_* has been estimated in section I. A pumping spot size R = 50 μm has been considered here. *W_k_^in^* is the rate of X-P scatter into the LPB state, *W_k_^out^* is the rate of X-P scatter out of the LPB state which can written as :

(6)

where $M_{k}$ is the matrix element of the LPB, $\rho_{x}$ is density of state of the bare exciton, $\varepsilon_{k}^{lp}$ is the energy of the LPB and $T_{x}$ is the exciton reservoir temperature. $T_{x}$ will be determined by the heating process of exciton-polariton scattering, the cooling process of LO-phonon-exciton scattering and the reservoir will try to stay at the lattice temperature $T_{L}$.^4^ $W_{k\to k'}^{lp-LO}$are the scattering rates of polariton-polariton scattering and polariton-LO phonon scattering.^5^ The corresponding parameters in our simulation are exciton Bohr radius a_B_ = 18 Å, energy of LO-phonon E_LO_ =72 meV. The exciton reservoir decay rate, Γ, is combined with radiative losses and exciton-exciton scattering loss (*P-band*). It is also worth mentioning that thermal broadening influence to exciton and LO-phonon is also included in the simulation.

Fig. S2a and S2b represent the calculated threshold tendency from 77K to 353K. The simulation results show a good consistency with measured data shown in Fig. 2 and Fig. 5a in the main manuscript. At low temperature, threshold shows two optimum dip due to the efficient phonon scattering assistance, as shown in Fig. S2a. The assistance from LO phonon scattering soften gradually with increasing temperature due to the thermal broadening effect, as shown in Fig. S2b. Above 300K, the LO-phonon assisted relaxation is no longer observed in our simulation, the threshold dependency is determined by the thermodynamics and kinetics.


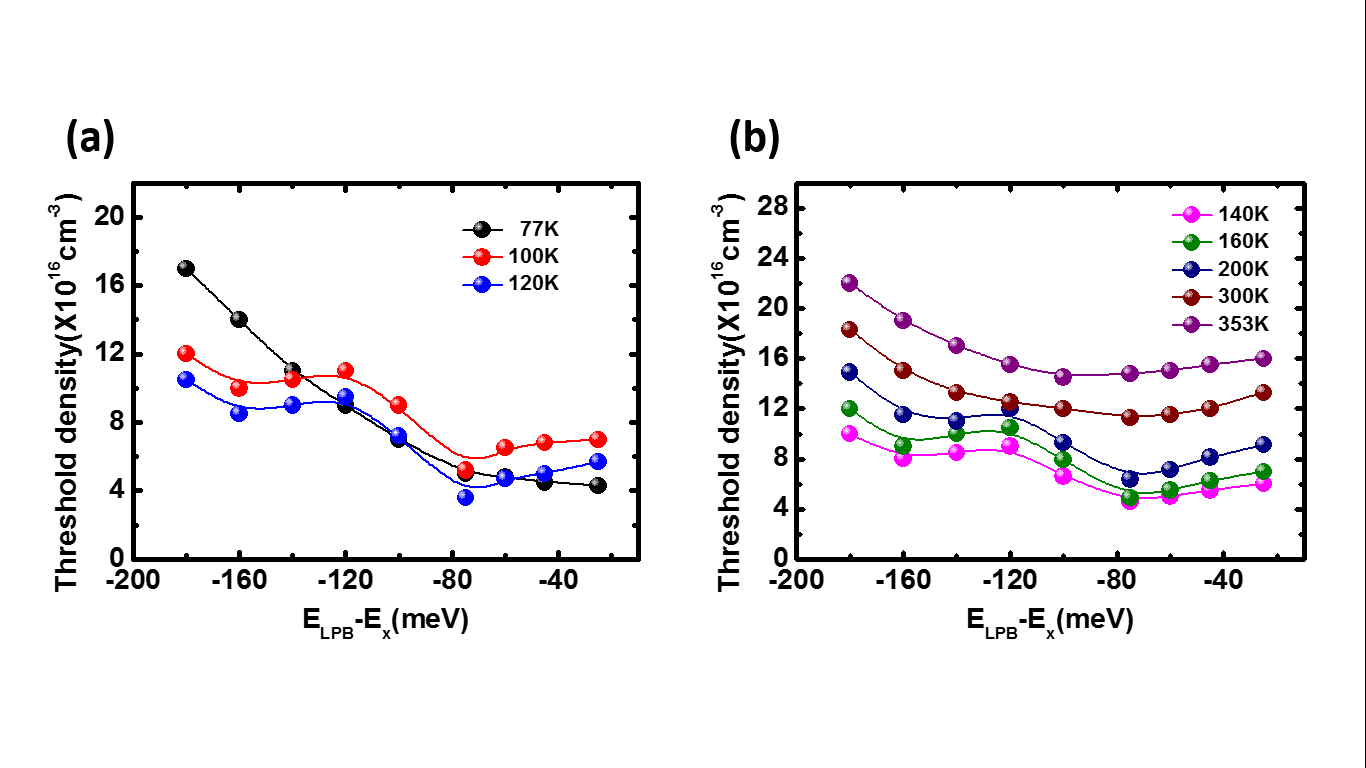


**Supplementary Figure S2.** Simplified phase diagram of a strong coupled ZnO MC. **a,** Polariton lasing threshold versus LPB-exciton offset at the temperature ranging from 77 K to 120 K. **b,** Polariton lasing threshold versus LPB-exciton offset at the temperature ranging from 140 K to 353 K.

**III. Characteristics of *P-band* exciton emission**

1. *Angular dispersion*

From Figs. 4a to 4c in the main manuscript, *P-band* exhibits a strongest emission intensity at 40 degree collection angle. This is because only the large angle part could be coupled out through polariton reflection dip since the cavity dispersion is modulated by the strong exciton-photon coupling. Therefore, the strongest P-band lasing angle would be varied by different cavity detunings. To verify this phenomena, we calculated the angle-resolved reflectivity of Fig. 4c using transfer matrix method accompanied with the exciton Lorentz oscillator model, as shown in Fig. S3a. The white dash line in Fig. S3a represent the fitted exciton (x), cavity photon (c), and LPB, which have the same parameters as in the Fig. 4c. It’s obvious that *P-band* emission is capped by DBR stopband and hard to couple out of the cavity. A more clear analysis is shown in Fig. S3b. At zero degree, the PL (blue solid line) shows a strong peak at the reflection dip (red solid line) and another weak emission at 3.29 eV which represent the onset polariton lasing and the P-band lasing, respectively. The weaker P-band lasing emission is attributed to the high reflectivity of the MC. For 40 degree, the cavity dip shifts toward 3.29 eV so that *P-band* lasing could couple out of the cavity efficiently.


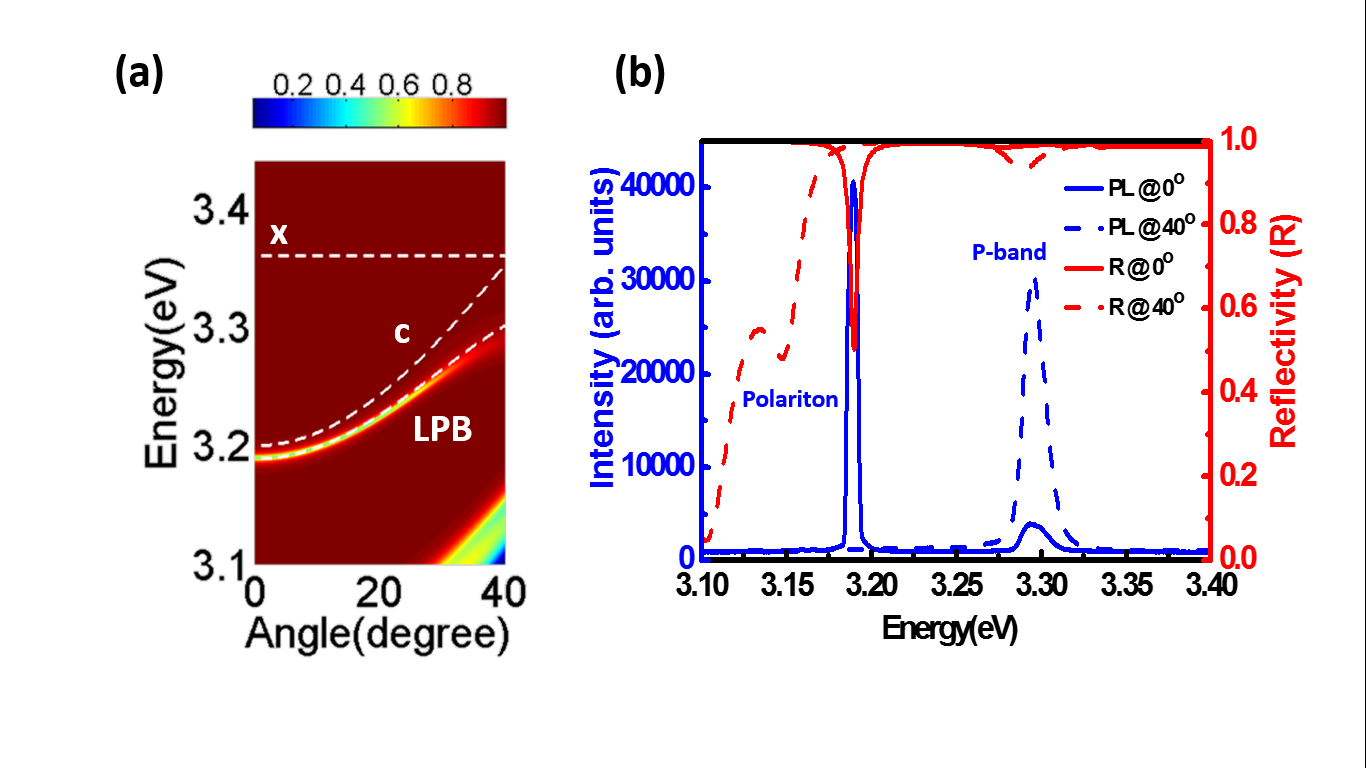


**Supplementary Figure S3.** Angle-resolved properties of P-band in a strong coupled ZnO MC. **a,** Calculated color map of ZnO MC reflectivity. **b,** Spectra of measured PL emission and calculated cavity reflectivity at 0°and 40° collection angle.

1. *Temperature dependent properties*

The temperature dependence of *P-band* exciton is plotted in the Fig. S4. The emission energy of *P-band* exciton follows the Varshni formula since it is generated from the excitons and is not influenced by the artificial structure such as MC.^5^ Fig. S4 also depicts the thermal broadening of *P-band*. The linewidth of *P-band* is gradually modulated by the phonon induced homogeneous broadening with increasing the temperature. With this feature, the exciton-exciton scattering also becomes more inefficient due to the ever shortening of exciton lifetime. This can be one of the reasons that the *P-band* lasing operation is only observed at the temperature below 140K in our measurements. Another possible reasons include reabsorption of the P_2_ emission and the favoring of exciton-electron scattering would also be proposed to prevent the P-band lasing occurred at higher temperature.^5^


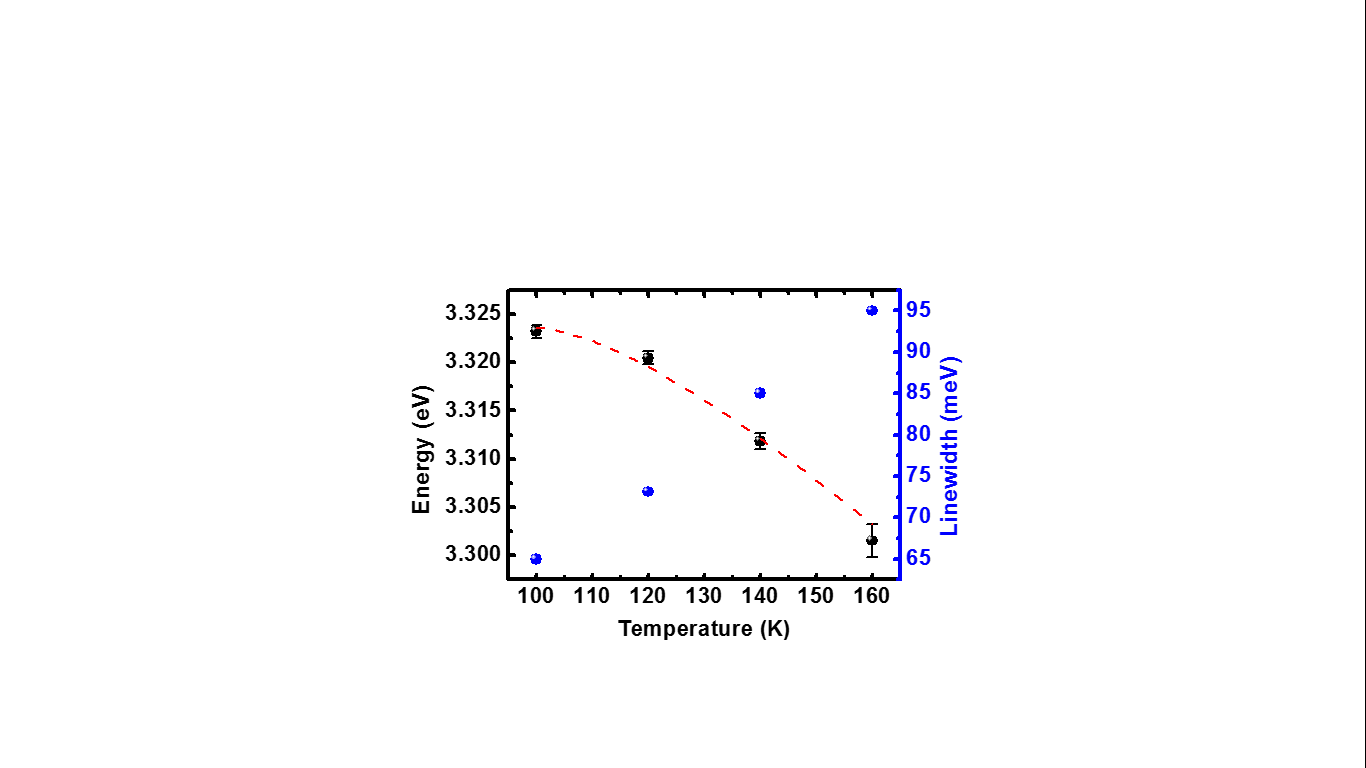


**Supplementary Figure S4.**Temperature dependence of *P-band* exciton. Linewidth and emission energy of *P-band* exciton versus temperature. The red dashed line indicates the Varshni law fitting curve.

**IV. Characteristics of polariton lasing at different temperature**


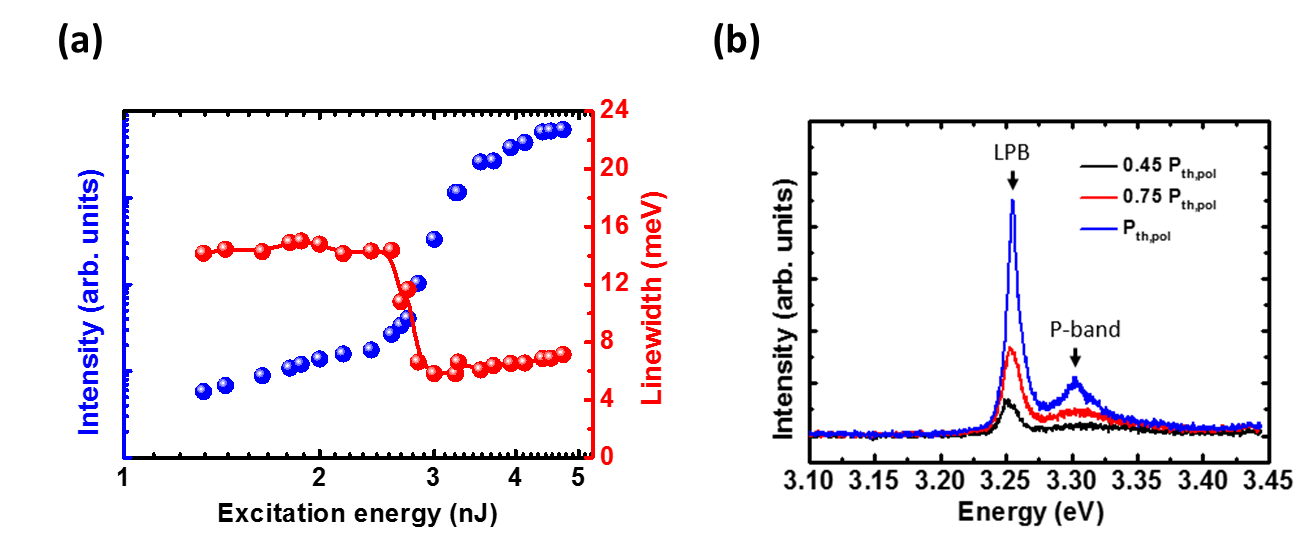


**Supplementary Figure S5.** Polariton lasing characteristics at 100K at detuning = -60 meV, corresponding to the case shown in Fig. 3e in the main text. **a.** Polariton peak intensity and linewidth of polariton emission versus excitation energy measured at the detection angle = 0°. **b.** The emission spectra below the polariton lasing threshold measured at the detection angle = 0°. In this case, only the polariton exhibits the lasing action and the P-band does not show stimulated emission.


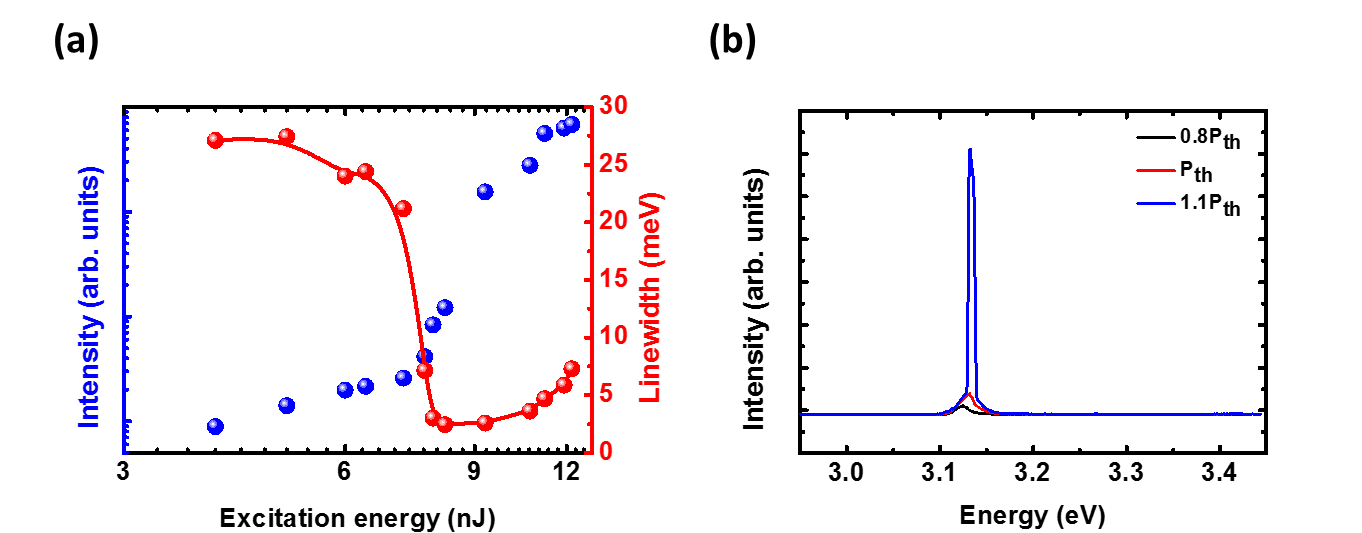


**Supplementary Figure S6.** Polariton lasing characteristics at 300K at E_LPB_-E_x_ = -150 meV. **a.** Polariton peak intensity and linewidth of polariton emission versus excitation energy measured at the detection angle = 0°. **b.** The polariton emission spectra below and above threshold measured at the detection angle = 0°.


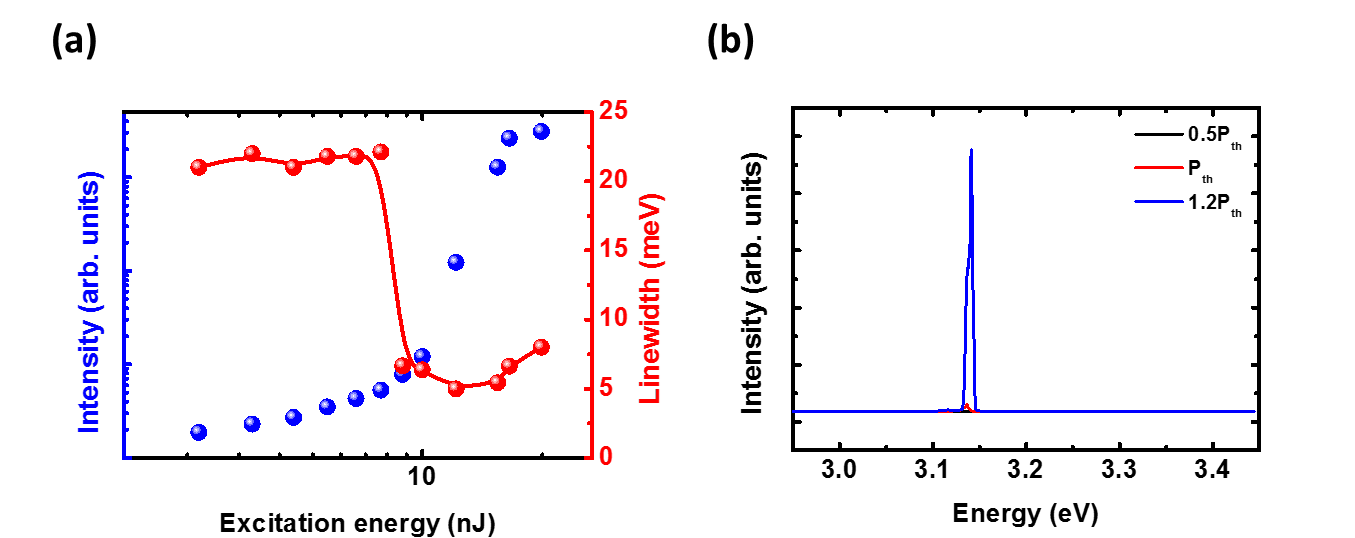


**Supplementary Figure S7.** Polariton lasing characteristics at 353K at E_LPB_-E_x_ =-138 meV. **a.** Polariton peak intensity and linewidth of polariton emission versus excitation energy measured at the detection angle = 0°. **b.** The polariton emission spectra below and above threshold measured at the detection angle = 0°.

At 300K and 353K, we can clearly see the onset of threshold in intensities along with the narrowing of linewidth at threshold. We can also see the typical signatures of polariton lasing above threshold: linewidth broadening and small blue-shifted peaks (not shown here, one can check Fig. 3a and b in the main manuscript).

**Reference**

1. Orosz, L. *et al.* Fabrication and Optical Properties of a Fully-Hybrid Epitaxial ZnO-Based Microcavity in the Strong-Coupling Regime. *Appl. Phys. Express* **94,** 061103 (2009).
2. Johne, R., Solnyshkov, D. D. & Malpuech, G. Theory of exciton-polariton lasing at room temperature in ZnO microcavities. *Appl. Phys. Lett.* **93,** 211105 (2008).
3. Reynolds, D. C. *et al.* Time-resolved photoluminescence lifetime measurements of the Γ_5_ and Γ_6_ free excitons in ZnO. *J. Appl. Phys.* **88,** 2152 (2000).
4. Porras, D., Ciuti, C., Baumberg, J. J. & Tejedor, C. Polariton dynamics and Bose-Einstein condensation in semiconductor microcavities. *Phys. Rev. B* **66,** 085304 (2002).
5. Klingshirn, C., Hauschild, R., Fallert, J. & Kalt, H. Room-temperature stimulated emission of ZnO: Alternatives to excitonic lasing. *Phys. Rev. B* **75,** 115203 (2007).
